# 2C-B-Fly-NBOMe Metabolites in Rat Urine, Human Liver Microsomes and *C. elegans*: Confirmation with Synthesized Analytical Standards

**DOI:** 10.3390/metabo11110775

**Published:** 2021-11-12

**Authors:** Jitka Nykodemová, Anna Šuláková, Petr Palivec, Hedvika Češková, Silvie Rimpelová, Klára Šíchová, Tereza Leonhardt, Bronislav Jurásek, Kateřina Hájková, Tomáš Páleníček, Martin Kuchař

**Affiliations:** 1 Forensic Laboratory of Biologically Active Substances, Department of Chemistry of Natural Compounds, University of Chemistry and Technology Prague, Technická 5, 166 28 Prague, Czech Republic; nykodemj@vscht.cz (J.N.); palivecp@vscht.cz (P.P.); ceskovah@vscht.cz (H.Č.); jurasekb@vscht.cz (B.J.); katerina1.hajkova@vscht.cz (K.H.); 2 Department of Experimental Neurobiology, National Institute of Mental Health, Topolová 748, 250 67 Klecany, Czech Republic; anna.sulakova@nudz.cz (A.Š.); klara.sichova@nudz.cz (K.Š.); tomas.palenicek@nudz.cz (T.P.); 3 Department of Biochemistry and Microbiology, University of Chemistry and Technology Prague, Technická 3, 166 28 Prague, Czech Republic; pikalovt@vscht.cz

**Keywords:** 2C-B-Fly-NBOMe, LC–MS, metabolite synthesis, metabolomics, human liver microsomes, *Cunninghamella elegans*, in vivo experiment (rats)

## Abstract

Compounds from the *N*-benzylphenethylamine (NBPEA) class of novel psychoactive substances are being increasingly utilized in neurobiological and clinical research, as diagnostic tools, or for recreational purposes. To understand the pharmacology, safety, or potential toxicity of these substances, elucidating their metabolic fate is therefore of the utmost interest. Several studies on NBPEA metabolism have emerged, but scarce information about substances with a tetrahydrobenzodifuran (“Fly”) moiety is available. Here, we investigated the metabolism of 2-(8-bromo-2,3,6,7-tetrahydrobenzo[1,2-b:4,5-b’]difuran-4-yl)-*N*-(2-methoxybenzyl)ethan-1-amine (2C-B-Fly-NBOMe) in three different systems: isolated human liver microsomes, *Cunninghamella elegans* mycelium, and in rats in vivo. Phase I and II metabolites of 2C-B-Fly-NBOMe were first detected in an untargeted screening and identified by liquid chromatography–tandem mass spectrometry (LC–MS/MS). Several hypothesized metabolites were then synthesized as reference standards; knowledge of their fragmentation patterns was utilized for confirmation or tentative identification of isomers. Altogether, thirty-five phase I and nine phase II 2C-B-Fly-NBOMe metabolites were detected. Major detected metabolic pathways were mono- and poly-hydroxylation, *O*-demethylation, oxidative debromination, and to a lesser extent also *N*-demethoxybenzylation, followed by glucuronidation and/or *N*-acetylation. Differences were observed for the three used media. The highest number of metabolites and at highest concentration were found in human liver microsomes. In vivo metabolites detected from rat urine included two poly-hydroxylated metabolites found only in this media. Mycelium matrix contained several dehydrogenated, *N*-oxygenated, and dibrominated metabolites.

## 1. Introduction

**Scheme 1:**
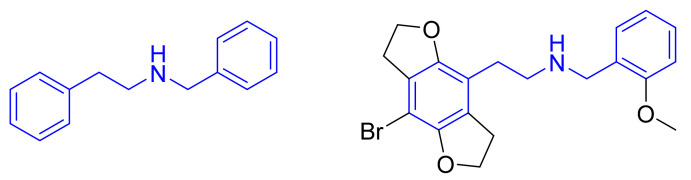
The chemical structure of NBPEAs and 2C-B-Fly-NBOMe (**1**).

New Psychoactive Substances (NPS) are novel analogues of already legally regulated psychoactive substances. NPS represent a very diverse set of compounds, which are divided into nine different groups based on their chemical structure [1]. One of these groups are *N*-benzylphenethylamines (NBPEAs, Scheme 1), highly potent agonists of the serotonin 2A receptor (5-HT2AR), originating from the phenethylamines by functionalization of the free amino group by a substituted benzyl moiety [2,3]. NBPEAs represent an extensive, diverse, and widespread group of psychedelic compounds that have gained large attention for recreational, therapeutic, and scientific uses [3]. Understanding the metabolism of these substances is of high interest from both pharmacological and toxicological perspectives.

2-(8-Bromo-2,3,6,7-tetrahydrobenzo[1,2-b:4,5-b’]difuran-4-yl)-*N*-(2-methoxybenzyl)ethan-1-amine (2C-B-Fly-NBOMe, compound **1**; Scheme 1), a substance belonging to the NBPEAs, was first reported in a 1999 poster presenting the results of a search for potent and stereoselective 5-HT2AR antagonists and its new agonists as tools for the study of 5-HT2AR-mediated functions [4]. The substitution of amine by a benzyl group with a methoxy (NBOMe) or hydroxy (NBOH) substituent in the *ortho* position leading to an extreme increase in potency at the 5-HT2 receptors was found to be generalizable [5,6]. This scaffold was further investigated by Ralf Heim, developing and analyzing the plethora of novel *N*-benzylphenethylamine analogues [7]. *N*-benzyl substitution of the phenethylamine exerts not only high binding affinity at the 5-HT2AR and serotonin 2C receptor (5-HT2CR) [2,3,8], but often also high selectivity over other serotonin receptor subtypes, that make these compounds intriguing potential tools to study 5-HT2AR-mediated functions. Compound **1** exhibits subnanomolar affinity and high activation efficacy of the 5-HT2AR in a neuronal GF62 cell line (K_i_ = 0.16 ± 0.04 nM with antagonist [^3^H]MDL100907; ED_50_ = 1.06 ± 0.19 nM assessed as effect on phosphoinositide (PI) hydrolysis; intrinsic activity 83% compared to the activation by 10 μM 5-HT) [9]. However, examination of in vivo biodistribution of compound **1** in pig brain indicated faster kinetics and reversibility of binding, therefore, it was not further examined as a suitable diagnostic tool [9]. Regardless of the inconvenient properties for pharmacy, such a high compound potency on these receptors may arouse the interest of psychonauts who explore the altered state of consciousness using psychedelic drugs.

In addition to the cardiotoxic [10] and neurotoxic [11,12] effects on cell lines, NBOMes can affect the cardiovascular system through inhibition of the human Ether-à-go-related Gene (hERG) potassium channel [10] and are further associated with intoxications with numerous seizures. In more severe cases these seizures may result in fatalities with symptoms such as acute kidney or even multiorgan failure [13]. Due to the high potency of these substances and relatively frequently occurring acute intoxications, which is in contrast with other psychedelics, these substances pose a serious health risk to the user. Their danger may be further exacerbated by their presence in counterfeit ecstasy or lysergic acid diethylamide (LSD) products [14,15,16]. Although an NBOMe intoxication is usually treated with symptomatic treatment, in many cases it is necessary to know the causes of individual poisoning and, therefore, it is necessary to have appropriate methodologies for non-target screening available. Such methods need to be sensitive enough due to the low concentrations of the parent substance in body fluids, which in some cases may already be below the limit of detection [17]. In such cases, the analysis must rely on the identification of metabolites, which may be responsible even for some of the toxic effects [18]. Knowledge of the metabolism of these substances and synthetic approaches for the preparation of these standards thus become relatively essential for laboratories focused on forensic toxicology.

**Scheme 2:**
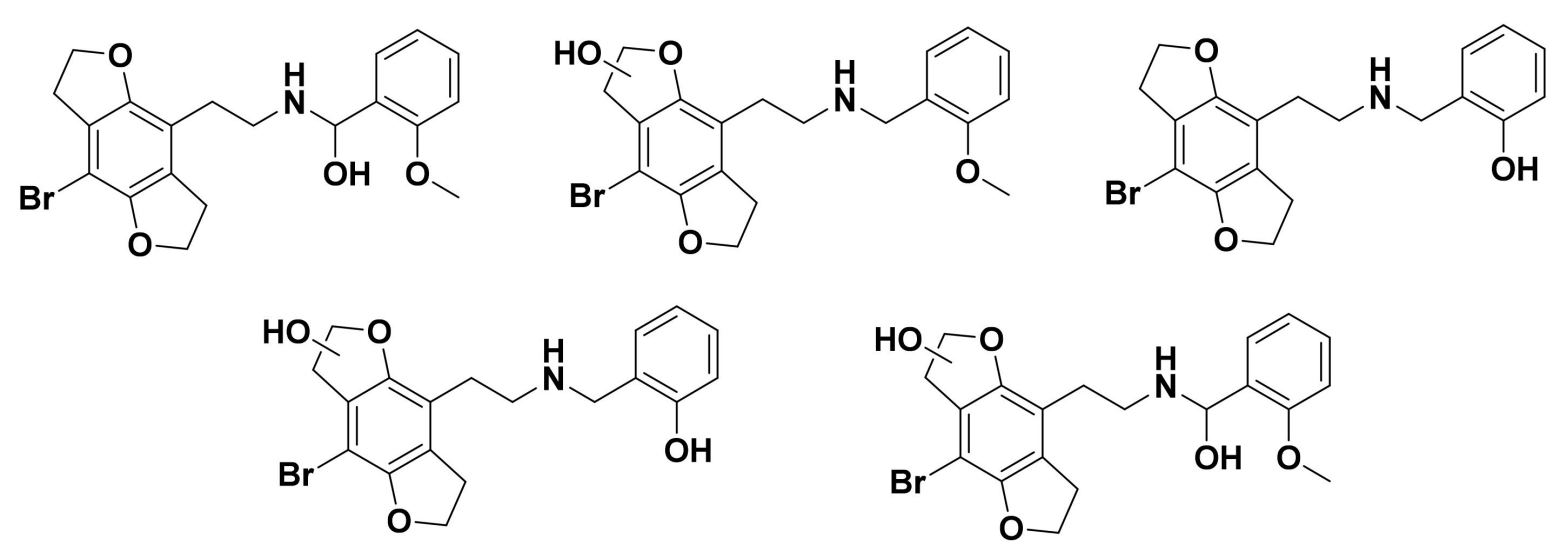
Tentative metabolites of compound **1** as reported by Richter et al. [19].

Since no data for 2C-B-Fly-NBOMe activity in humans are currently available, Richter et al. [19] studied the metabolism of 2C-B-Fly-NBOMe in terminally differentiated human cells from hepatocellular carcinoma (HepaRG), identifying in a total of five phase I metabolites and one phase II metabolite. Two mono-hydroxylated isomers, one *O*-demethylated and one *O*-demethylated-mono-hydroxylated metabolites were tentatively identified (see Scheme 2). The 2C-B-Fly-NBOH metabolite further undergoes conjugation with glucuronic acid, providing 2C-B-Fly-NBOH glucuronide, which is the only phase II metabolite identified in this study [19]. However, since no standards were used in the metabolic study, the exact positions of the hydroxy moieties of the described hydroxylated metabolites remain unclear.

Because only five metabolites of 2C-B-Fly-NBOMe were known, we aimed to identify other metabolites of this compound. In the present study, we focused on the comparison of the metabolism of 2C-B-Fly-NBOMe in human liver microsomes (HLM), in the mycelium of *Cunninghamella elegans* (*C. elegans*), and in vivo in rats. Pooled HLM are both a verified and cheap system for studies of the metabolism of compounds. Not only do they well represent a true population sample, but also are fully characterized for cytochrome P450 activities and selected phase II enzymes. *C. elegans* is also a cheap and reliable system that can be used for metabolism studies as it can facilitate reactions such as *N*-dealkylation, hydroxylation, or dehydrohalogenation. Rat urine is often used as a golden standard in forensic science for metabolism studies. We aimed to compare the differences between our in vitro and in vivo models, as well as confirm or exclude proposed metabolites by comparison with synthesized standards. First, a non-targeted screening was performed using HPLC with a high-resolution mass detector. We synthesized and fully characterized standards of several proposed metabolites and deuterium-labeled standards 2C-B-Fly-NBOMe-*d*_3_ and 2C-H-Fly-NBOMe-*d*_3_ and used them to confirm or disprove their occurrence.

## 2. Results

### 2.1. Untargeted Analysis and Synthesis of Reference Standards

For the identification of metabolites, an untargeted screening approach was applied. First, high-performance liquid chromatography (HPLC) on a reversed-phase was employed for the separation of HLM, *C. elegans* growth medium, and rat urine samples. A hybrid triple quadrupole time-of-flight mass spectrometer was utilized for high-resolution measurements. The obtained data were screened for exact precursor ion masses (PMs) of presumable metabolites. High-resolution tandem mass spectrometry (HR-MS/MS) data were recorded as well (Table S1 in Supplementary Data). For unambiguous identification of the formed metabolites and a better understanding of their fragmentation patterns, we opted to synthesize tentative metabolites formed by hydroxylation, *O*-demethylation, debromination, *N*-demethoxybenzylation, and combinations thereof.

**Scheme 3:**
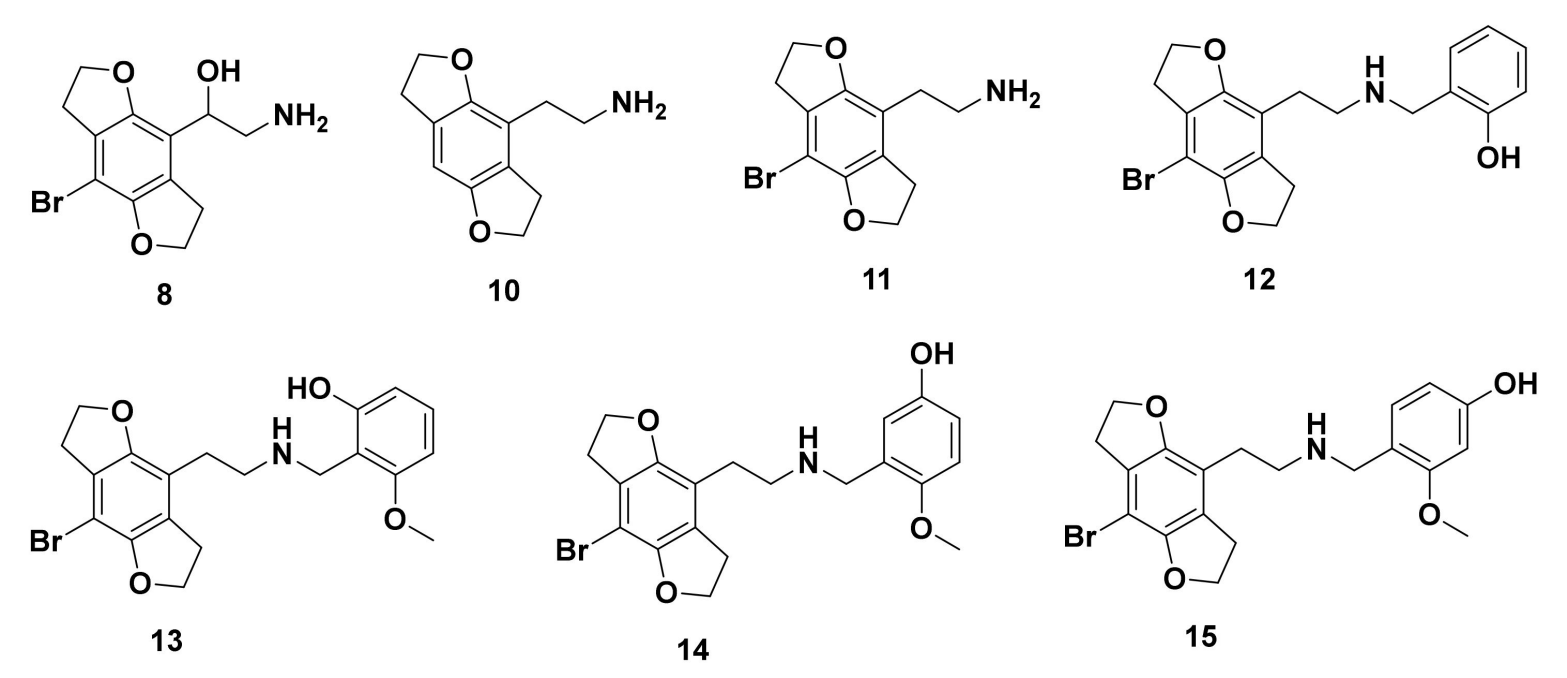
Synthesized metabolites **8**, **10**–**15**.

**Scheme 4:**
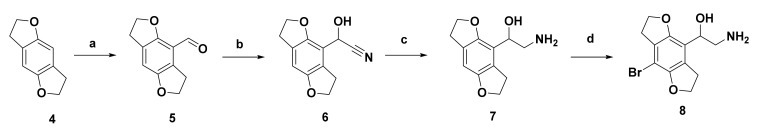
Synthesis of *β*-hydroxy-2C-B-Fly (**8**). (**a**) DCMME, SnCl_4_; (**b**) KCN, H_2_SO_4_; (**c**) LiAlH_4_; (**d**) Br_2_, AcOH.

A key intermediate in the synthesis of “Fly” compounds is the heterocyclic nucleus, tetrahydrobenzo[1,2-b;4,5-b’]difuran (**4**), which was synthesized by previously published procedures [20,21] starting from commercially available 1,4-bis(2-hydroxyethoxy)-benzene. Formylation, Henry condensation to nitroalkene, its reduction to phenethylamine by lithium aluminum hydride provided 2C-H-Fly (**10**; Scheme 3), and lastly, aromatic bromination provided 2C-B-Fly (**11**). Metabolites bearing hydroxy group on the *N*-benzyl moiety (**12**–**15**) were prepared by reductive alkylation of compound **2** with commercially available methoxy-hydroxybenzaldehydes. *β*-hydroxy derivative (**8**; Scheme 3) was prepared as depicted in Scheme 4. Hydroxynitrile **6** was formed by cyanohydrin reaction and separated from the aldehyde **5** by column chromatography, subsequently reduced to the *β*-hydroxy-phenethylamine (**7**) by lithium aluminum hydride and finally brominated by elemental bromine in acetic acid. Due to synthetic difficulty, standards for metabolites bearing hydroxy groups on the furan rings were not attempted. Debrominated metabolite (16) was prepared by reductive alkylation of **10**.

### 2.2. LC–MS Analysis of the Synthesized Standards of Proposed Metabolites

All authentic standards of the proposed metabolites prepared by organic synthesis were subjected to a thorough analysis of first- and second-order fragmentation. The acquired data are summarized in Table 1 and the origin of characteristic fragment ions (FIs) is clarified below.

### 2.3. Detection of Metabolites

Both HR-MS only and HR-MS/MS data were assessed concerning the observed fragmentation patterns of in-house synthesized reference standards. Identification of 2C-B-Fly-NBOMe metabolites was based on the recorded exact masses of precursor ions (PMs) and their characteristic fragment ions (FIs) in high-res-MS^2^. Table 2 summarizes all detected metabolites.

Obtained information-dependent analysis (IDA) scans were evaluated in agreement with the standard screening approaches conventions. Most abundant product ions, listed in Supplementary Table S1, were used to propose the chemical structures of individual metabolites. For unambiguous identification of the metabolite, the accurate mass of precursor ion must match the calculated monoisotopic mass and the underlying HR-MS/MS spectrum must fit with a library spectrum of the reference standard. In the absence of available reference spectra, at least the major peaks in recorded HR-MS/MS spectra were assigned to particular fragment ions for tentative identification of metabolites, even though it was not always possible to determine the exact positions of functional groups. For detection, the accurate mass of precursor ion and its isotope pattern had to match with the proposed elemental composition. Altogether, thirty-five phase I and nine phase II metabolites of 2C-B-Fly-NBOMe were detected.

Concerning phase II metabolites, seven *O*-glucuronides and two *N*-acetylated metabolites were detected. Their fragmentation patterns are in agreement with the corresponding phase I metabolites after elimination of glucuronic acid (–C_6_H_8_O_6_; −176.0321 u) in the case of glucuronides of the acetyl moiety (–C_2_H_2_O; −42.0106 u) in the case of *N*-acetylated compounds. Several hydroxy metabolites (M9-G, M19-G, M22-G, and M-Ac2) lose water (–H_2_O; −18.0106 u) at the same time.

## 3. Discussion

The molecules were conceptually divided into two distinct parts, i.e., the 2,3,6,7-tetrahydrobenzo[1,2-b:4,5-b’]difuran-4-yl)ethanamine (Fly) part and the *N*-(2-methoxybenzyl) (NBOMe) part. Observed fragmentation patterns of 2C-B-Fly-NBOMe (**1**; M1; PM at *m*/*z* 404.0861, M + H) are in good agreement with previously published data in [9]. The major FIs correspond to the cleavage of the methoxybenzyl moiety (at *m*/*z* 121.0653) and tropylium ion (at *m*/*z* 91.0548) formed after the loss of the methoxy group (−30.0106 u), as is the case with metabolites bearing the unchanged NBOMe part. The FI at *m*/*z* 325.1678 results from a loss of bromine as a radical. Previously undescribed FI detected at *m*/*z* 296.1412 could not be formed from the unchanged parent compound **1**, instead, it probably arose from a loss of ammonia following a rearrangement reaction initiated by an intramolecular electrophilic attack of the benzyl carbon at the tetrahydrobenzodifuran ring system, analogous to a rearrangement postulated by Caspar et al. [22] for 2-(4-iodo-2,5-dimethoxyphenyl)-*N*-(2-methoxybenzyl)ethanamine (25I-NBOMe). The FIs representing the Fly part showed a low abundance of less than 8%.

The situation changes dramatically with *O*-demethylation at the NBOMe part, as represented by fragmentation of 2C-B-Fly-NBOH (**12**; M6; PM at *m*/*z* 390.0705, M + H). FIs formed from the Fly part were much more abundant, presumably due to a hydrogen bond formation between the nitrogen and the hydroxy group. The FIs registered at *m*/*z* 284.0286, 267.0021, and 188.0837 correspond to protonated 2C-B-Fly (**11**), an ion formed by a loss of ammonia (−17.0266 u) and by the subsequent loss of bromine as a radical, respectively. Cleavage of hydroxybenzyl moiety resulted in the FI at *m*/*z* 107.0497.

Higher abundances of FIs representing the Fly part were also noticeable with derivatives bearing an extra hydroxy group at the NBOMe part. We synthesized three tentative monohydroxylated metabolites of 2C-B-Fly-NBOMe (PM at *m*/*z* 404.0861, M + H), the only difference between them being the exact position of the hydroxy group attached to the aromatic ring of NBOMe moiety. For all of them, the most abundant FIs corresponds to the cleavage of the modified NBOMe moiety (at *m*/*z* 137.0603) and to subsequently formed tropylium ion with the attached hydroxy group (at *m*/*z* 107.0497). We consider the intramolecular hydrogen bridge formation between the nitrogen atom and the C6’-hydroxy group to be the cause of increased abundance of the Fly part fragments and of the prolonged retention time of C6′-hydroxy-2C-B-Fly-NBOMe (**13**) compared to other studied isomers. In addition to the typical Fly part FIs (at *m*/*z* 284.0286, 267.0021, and 188.0837), C5’-hydroxy-2C-B-Fly-NBOMe (**14**) also provided fragments resulting from a loss of bromine (FI at *m*/*z* 341.1627), and the aforementioned rearrangement followed by the amine shift (FI at *m*/*z* 312.1362).

Fragmentation patterns of 2C-H-Fly-NBOMe (M7; PM at *m*/*z* 326.1756, M + H) were examined using the deuterated analogue of the compound with three deuterium atoms bound in the methoxy group (**16**). As the NBOMe part of the molecule remained unsubstituted, the major FIs correspond to the cleavage of the methoxybenzyl group (at *m*/*z* 121.0653) and subsequently formed tropylium ion (at *m*/*z* 91.0548). The FI at *m*/*z* 309.1491 resulted from a loss of ammonia (−17.0266 u) following the same rearrangement reaction described for 2C-B-Fly-NBOMe (**1**). The Fly part was represented by the iminium ion (at *m*/*z* 204.1025) formed by benzyl cleavage, and the FI at *m*/*z* 189.0916 formed by the subsequent loss of NH (−15.0109 u). Indeed, the only difference between the latter ion and the Fly part FI (at *m*/*z* 188.0837) typical for 2C-B-Fly (M8; PM at *m*/*z* 284.0286, M + H) and other brominated Fly compounds is the presence of a hydrogen atom at position 8 of the 2,3,6,7-tetrahydrobenzo[1,2-b:4,5-b’]difuran ring system.

One monohydroxylated 2C-B-Fly isomer (**8**, PM at *m*/*z* 300.0235, M + H) carrying the hydroxy group in benzylic position was synthesized and used to study the fragmentation fate of such compounds hydroxylated at the Fly part of the molecule. The major FI (at *m*/*z* 203.0946), also formed from FI (at *m*/*z* 282.0130) representing dehydrated (–H_2_O; −18.0106 u) parent compound **8**, corresponds to unsaturated ammonium ion arising from a loss of bromine. The presence of a double bond resulting from water elimination is evident in other registered FIs (at *m*/*z* 264.9864 and 186.0684) as well. However, these fragments cannot be used to determine the exact position of the hydroxy group within the Fly part.

From the synthesized metabolites, 2C-B-Fly-NBOMe (**1**; M1), 2C-B-Fly-NBOH (**12**; M6), 2C-H-Fly-NBOMe (**16**; M7), 2C-B-Fly (**11**; M8), and C5’-hydroxy-2C-B-Fly-NBOMe (**14**; M2) were confirmed in experimental samples. The other three monohydroxylated metabolites (M3–M5) have the unchanged methoxyarene ring (typical FIs at *m*/*z* 121.0653 and 91.0548) and bear the hydroxy group at sp3 hybridized carbon atom either in the Fly part (unsaturated FI at *m*/*z* 264.9864) or in the benzylic position of NBOMe part of the molecule (FI at *m*/*z* 267.0021 suggests intact Fly part). Although we proposed and synthesized an *N*-demethoxybenzylated-monohydroxylated metabolite (**8**), it was not the isomer found in our analysis. The detected metabolite is hydroxylated at the 2,3,6,7-tetrahydrobenzo[1,2-b:4,5-b’]difuran; however, the exact position remains unknown. Characteristic Fly and NBOMe part FIs recognized in preceding fragmentation analysis were searched for in HR-MS/MS data to determine the structure of the preliminarily identified metabolites as accurately as possible.

Among polyhydroxylated substances, dihydroxylation of the Fly part could be distinguished by FIs at *m*/*z* 279.9973 and 262.9708 representing protonated Fly part with two double bonds and the compound formed by a subsequent loss of ammonia (−17.0266 u), respectively. Several dehydrogenated metabolites were also tentatively identified. However, if both a hydroxy group and a double bond are present in the metabolite’s structure, it is virtually impossible to propose the exact position of these functional groups without comparison with a reference standard, as the same FIs (at *m*/*z* 282.0130 and 264.9864) indicate the presence of both of these structural features in the Fly part. Oxidative debromination provides phenolic compounds bearing the hydroxy group at position 8 of the 2,3,6,7-tetrahydrobenzo[1,2-b:4,5-b’]difuran ring system. These substances form the FI at *m*/*z* 151.0759 corresponding to the core heterocyclic nucleus still carrying the phenolic hydroxy group after the cleavage of one of the tetrahydrofuran rings with only a methyl group remaining at the benzene ring.

### 3.1. Comparison of 2C-B-Fly-NBOMe Metabolism in Different Species

Diverse results of metabolic transformations in vivo in rats and in vitro in *C. elegans* mycelium and HLM were observed. Incubation of microsomes was generally rich in phase I metabolites with twenty-nine metabolites detected. The concentration levels of confirmed metabolites were also the highest in this matrix.

Directly after subcutaneous application of a relatively high dose (50 mg·kg^−1^) of 2C-B-Fly-NBOMe (**1**) into the rats, their urine was continuously collected in a metabolic cage. Two portions of urine were collected after 6 h and subsequently after 24 h to identify early phase and late phase metabolites. The dose was selected according to the potency of related compounds after it was proven in-house to be safe for in vivo experiments using Modified Acute Toxicity assessment (OECD no. 423, 2001; unpublished data). Rat urine samples contained fifteen phase I metabolites. Three of them—one isomer of dihydroxylated metabolite (M12) and two demethylated compounds hydroxylated to higher degrees (M24, M25)—were identified as urinary metabolites only, as they were not formed in in vitro protocols. On the other hand, the overall number and concentration level of urinary metabolites were lower compared to incubation of HLM. Altogether, nine phase II metabolites were also excreted in rat urine, of which seven were conjugates of glucuronic acid.

*C. elegans* released to the medium seventeen metabolites. Apart from those formed by the most widespread transformations of NBPEAs (hydroxylation, *O*-demethylation, *N*-demethoxybenzylation), several dehydrogenated metabolites (M26–M30 and M32) and even two *N*-oxides were produced by the mycelium. The extent of debromination leading to 2C-H-Fly-NBOMe (M7) was highest in this matrix. In one case, *C. elegans* generated an isomer hydroxylated in a different position (M18) compared to that found in rat urine and microsomal incubation.

### 3.2. Proposed Metabolic Pathways

**Figure 1:**
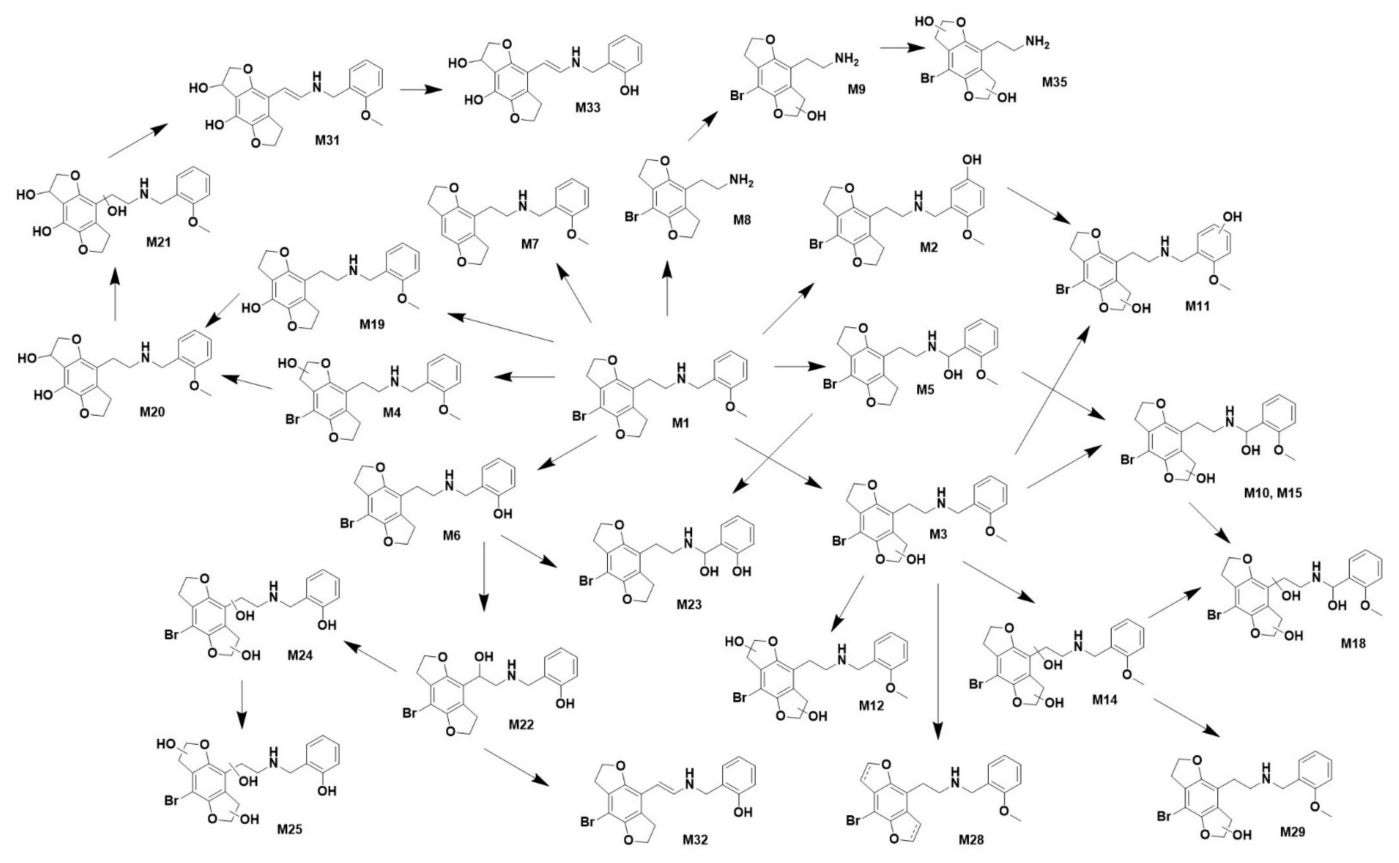
Metabolic pathways of 2C-B-Fly-NBOMe.

The metabolic pathways of 2C-B-Fly-NBOMe (M1) are depicted in Figure 1. They were propounded based on the metabolites observed either in rat urine, HLM, or *C. elegans* mycelium. The parent drug M1 undergoes the same major metabolic transformations described for structurally related *N*-benzylphenethylamines, for example, 2C-B-NBOMe [23]. The heterocyclic tetrahydrobenzo[1,2-b;4,5-b’]difuran core remains unopened but represents the preeminent site for hydroxylation and subsequent dehydration leading to restoration of aromaticity of the furan ring. The same metabolic fate of the core heterocyclic structure has been described for several unsubstituted Fly compounds including 2C-B-Fly, 2C-E-FLY, 2C-EF-FLY, and 2C-T-7-FLY [24,25]. In good agreement with previously reported data [19], the most abundant metabolites are the result of hydroxylation, dihydroxylation, *O*-demethylation, and a combination thereof. *N*-Demethoxybenzylation represents only a minor metabolic pathway.

The following transformations were found to give rise to phase I metabolites: hydroxylation (M2, M3, M4, M5), dihydroxylation (M10, M11, M12, M13, M14, M15), and trihydroxylation (M17, M18), demethylation (M6), *N*-demethoxybenzylation (M8), debromination (M7), a combination of demethylation with monohydroxylation or polyhydroxylation (M22, M23, M24, M25), and combination of *N*-demethoxybenzylation with hydroxylation or dihydroxylation (M9, M35).

Even though the debrominated metabolite M7 was found in *C. elegans* culture media and also in HLM samples in a limited amount, it seems that the preferred way of bromine cleavage is oxidative debromination resulting in corresponding phenolic compound (M19, M20, M21, M31, M33, and M34). Furthermore, if the Fly part is substituted with an extra hydroxy group, the retention time of such a phenolic analyte is usually high, probably due to intramolecular hydrogen bridge bond formation between the two hydroxy groups, although the eventuality of oxidation to *N*-oxide cannot be completely ruled out without comparison with an authentic standard.

Several dehydrogenated metabolites (M26–M34) were observed predominantly in HLM samples. Dehydrogenation was described as a CYP-catalyzed reaction for the 2C-NBOMe drug family [26]. According to our study, the compounds with newly formed double bonds could further be metabolized by hydroxylation (M29), hydroxylation and oxidative debromination (M31), *O*-demethylation (M32), and combination thereof (M33, M34). On the other hand, we bring evidence that a dehydrogenated metabolite could also be formed by artificial dehydration of the corresponding metabolite hydroxylated at the α-position to the nitrogen, as the unstable hemiaminal eliminates water in the ion source of the mass spectrometer. The formation of such an artificial metabolite was predicted by Caspar et al. [23] and is supported by our observation that metabolite M26 accompanies its hydrated counterpart—hydroxylated metabolite M5, at the same retention time, and is present in the same experimental samples.

In rat urine samples, several phase II metabolites were formed by glucuronidation (G) of the corresponding phase I metabolites (M3-G, M4-G, M9-G, M11-G, M19-G, M22-G1, M22-G2). Two *N*-acetylated metabolites (M-Ac1, M-Ac2) were detected only in their conjugated forms.

## 4. Materials and Methods

### 4.1. Human Liver Microsomes

To study the 2C-B-Fly-NBOMe metabolism, isolated HLM pooled from fifty donors (Thermo Fisher Scientific, Waltham, MA, USA) were chosen [19]. Upon delivery, the HLM were kept at −140 °C in the dark. For the experiment, HLM were thawed slowly on ice, which was followed by reaction mixture preparation. The mixture consisted of 2 µL of 2C-B-Fly-NBOMe (final concentration of 10 µM), 25 µL of HLM (20 mg·mL^−1^), and 163 µL of 100 mM phosphate-buffered saline. The reaction mixture was preincubated at 37 °C for 10 min in a water bath. Then, reaction initiation was started by the addition of 10 μL of 20 mM freshly prepared nicotinamide adenine dinucleotide phosphate (NADPH) dissolved in 0.1 M phosphate-buffered saline. After that, the reactions proceeded for 0, 15, 30, 45, and 60 min at 37 °C with gentle agitation. The reaction termination was performed using the addition of 200 µL of ice-cold acetonitrile at each time point. Directly after the reactions were quenched, the samples were frozen at −80 °C and kept until the MS analysis. As negative controls, we used: a no 2C-B-Fly-NBOMe sample, a no HLM sample, a no NADPH sample, and a heat-inactivated microsome sample (45 °C, 30 min pretreatment). Further, to verify unaffected HLM metabolism, as a positive control, 10 µM testosterone was used. The experiment was performed in two independent replicates.

### 4.2. Cunninghamella elegans

*C. elegans* mycelium (Cunninghamella elegans Lendner 10028b™) was maintained on potato dextrose (PD) agar plates (12 g·L^−1^ PD medium, 5 g·L^−1^ agar, both Formedium, UK) at 24 °C in the dark. For the experiment, a modified procedure according to Grafinger et al. [27] was used. Approx. 1 cm^2^ of the mycelium was scraped from a plate and inoculated onto a fresh PD agar plate, on which it was grown for 9 days at 24 °C in the dark. Then, approx. 1 cm^2^ of the freshly grown culture was inoculated (i) onto a fresh PD agar plate and (ii) into 35 mL of liquid PD medium in a 500 mL Erlenmeyer flask. PD agar plates with the inoculated mycelia were grown at 24 °C in the dark; PD medium with the inoculated mycelia was grown at 28 °C in the dark on a rotary shaker at 100 rpm. After 4 days of growth, the reaction with 2C-B-Fly-NBOMe was set up. First, 50 mg 2C-B-Fly-NBOMe was dissolved in 4 mL of distilled water and 2 mL of methanol and vortexed until the solution was clear. Then, 5 mL of this solution was mixed with 5 mL of PD medium and the mixture was filtered through a 0.22 µm polyvinylidene difluoride membrane filter to ascertain sterility. In a 50 mL sterile tube, 35 mL of liquid PD medium, 2 mL of the 2C-B-Fly-NBOMe + PD medium filtered mixture (or 2 mL of distilled water/methanol 2:1 mixture in the case of a control sample), and *C. elegans* mycelium scraped from the 4-day old PD agar plate were homogenized by vortexing for 1 min. The contents of the 50 mL tube were added to the 500 mL Erlenmeyer flask with the 4-day old mycelium, the flask was briefly mixed by swirling and 2 mL of the medium was sampled as the starting point of the reaction. Further samples were taken at 24, 48, 72, and 168 h. The samples were stored at −80 °C until the analysis. The total amount of 2C-B-Fly-NBOMe per reaction was 8.32 mg, i.e., the final concentration was 0.12 mg·mL^−1^. Control reactions with no 2C-B-Fly-NBOMe and with no mycelia were set up.

### 4.3. Animals

Urine samples of laboratory rats were obtained using an adapted method described earlier [28,29,30,31]. All used animals were Wistar rats (*n* = 7) acquired from VELAZ (Prague, Czech Republic), approximately 11 weeks old and weighing 300–375 g. Since oestrous cycle may affect metabolism in rats [28], only males were used. Animals were housed in pairs in a 12/12 h light/dark cycle with ad libitum water and food pellets. Both temperature (22 ± 2 °C) and humidity (30–70%) were controlled. 2C-B-Fly-NBOMe (**1**) was dissolved in deionized water and 20 μL of Tween-20 using an ultrasonic homogenizer. The compound was injected in a dose of 50 mg·kg^−1^ subcutaneously (s.c.) in a volume of 2 mL·kg^−1^. Urine samples were collected using metabolic chambers equipped with a pre-cooled collector maintaining the temperature of −4 °C for the following 24 h. The samples were subsequently frozen and kept at −80 °C until the analyses.

All experiments were conducted according to the principles of animal welfare of the National Committee for the Care and Use of Laboratory Animals (Prague, Czech Republic), and Guidelines of the European Union (86/609/EU). The protocol was approved by the National Committee for the Care and Use of Laboratory Animals (Prague, Czech Republic) under the number MZDR 48237/2017-3/OVZ on 9 October 2017.

### 4.4. Chemistry

Chemicals for the synthesis were purchased from Sigma-Aldrich. Air-sensitive operations were performed in dried solvents, which were dried using molecular sieves, and under an atmosphere of dry argon. The thin-layer chromatography (TLC Silica gel 60 F254) was used to monitor the reactions. Melting points were determined using a melting point apparatus (PGH Rundfunk-Fernsehen, Niederdorf, Germany) and are uncorrected. NMR spectroscopy was performed on Agilent 400 MR DDR2 (^1^H NMR 400 MHz, ^13^C NMR 100 MHz) at room temperature and the spectra were referenced on the residual peak of the solvents (CD_3_OD, CDCl_3_, or DMSO-d_6_). Flash Chromatography was performed on Combliflash Rf200 UV/VIS (Teledyne Technologies, California) using an in-house-made column that was filled with polar silica (Merck, Darmstadt, Germany).

**1,4-Bis(2-chloroethoxy)benzene (2)**

Synthesized as previously reported [20], ^1^H and ^13^C NMR spectra are in agreement with the literature [20].

**1,4-Bis(2-chloroethoxy)-2,5-dibromobenzene (3),**

**2,3,6,7-Tetrahydrobenzo[1,2-b:4,5-b’]difuran (4),**

**4-Formyl-2,3,6,7-tetrahydrobenzo[1,2-b:4,5-b’]difuran (5)**

Synthesized as previously reported [21], ^1^H and ^13^C NMR spectra in accordance with literature [21].

**2-Hydroxy-2-(2,3,6,7-tetrahydrobenzo[1,2-b:4,5-b’]difuran-4-yl)acetonitrile (6)**

To an ice-water bath cooled solution of aldehyde **5** (1.66 g, 8.73 mmol) and KCN (3.4 g, 52.63 mmol) in MeOH (200 mL), H_2_SO_4_ was carefully added dropwise until pH ~ 4. The reaction was then removed from cooling and stirred at room temperature overnight. The solvent was evaporated under reduced pressure, diluted in water, and extracted with DCM (2x). Organic phases were washed with NaHCO_3_, brine, dried over Na_2_SO_4_, filtered, and evaporated under reduced pressure to a yellow oil, that was purified by flash column chromatography on silica gel with a gradient elution of DCM to DCM with 5% EtOAc, recovering starting material **5** (0.26 g, 1.39 mmol, 16%) and the title product (1.59 g, 7.32 mmol, 84%).

**m.p.** 93–94.5 °C; **^1^H NMR** (400 MHz, CDCl_3_) δ 6.69 (s, 1H), 5.50 (d, *J* = 8.6 Hz, 1H), 4.71–4.62 (m, 2H), 4.60 (t, *J* = 8.7 Hz, 2H), 3.59 (d, *J* = 8.6 Hz, 1H), 3.35–3.18 (m, 2H), 3.17 (t, *J* = 8.6 Hz, 2H); **^13^C NMR** (101 MHz, CDCl_3_) δ 154.21 (s), 152.02 (s), 126.18 (s), 125.57 (s), 124.58 (s), 118.89 (s), 105.37 (s), 71.71 (s), 59.00 (s), 30.37 (s), 30.23 (s), 28.71 (s).

**2-Amino-1-(2,3,6,7-tetrahydrobenzo[1,2-b:4,5-b’]difuran-4-yl)ethan-1-ol (7)**

To an ice-water bath cooled suspension of LiAlH_4_ (700 mg, 18.41 mmol) in dry THF (8 mL) under a flow of argon was dropwise added a solution of cyanohydrin **6** (400 mg, 1.84 mmol) in dry THF (4 mL). The reaction was then removed from cooling and stirred at room temperature until full consumption of the starting material (TLC; DCM/MeOH/NH_4_OH 10:1:0.01, Rf(**7**) = 0.25). The reaction was thereafter cooled by an ice-water bath and quenched by the subsequent addition of a 1:1 mixture of THF and chilled water (0.8 mL), 15% NaOH (0.8 mL), and water (2.4 mL). Anhydrous MgSO_4_ was then added, and the reaction mixture was warmed up to room temperature. After stirring for 15 min, the suspension was suction filtered, filter washed thoroughly with THF, and the filtrate evaporated under reduced pressure. The residue was diluted in 1M HCl, washed with Et_2_O (2x), the aqueous phase was basified with 2 M NaOH to pH ~ 9 and extracted with DCM (5x). Combined DCM extracts were dried over Na_2_SO_4_ and evaporated under reduced pressure to crude white solid. The crude was purified by flash column chromatography using silica gel as the stationary phase and a gradual elution of DCM with 0.5% NH_4_OH to 10% MeOH in DCM with 2% NH_4_OH, providing the title compound as a white solid (238 mg, 1.07 mmol, 58%).

**^1^H NMR** (400 MHz, CDCl_3_, free base) δ 6.55 (s, 1H), 4.94 (t, *J* = 5.4 Hz, 1H), 4.57–4.46 (m, 4H), 3.53–3.46 (m, 1H), 3.28–3.21 (m, 2H), 3.18 (t, *J* = 7.6 Hz, 1H), 3.12 (t, *J* = 4.8 Hz, 2H), 2.95–2.90 (m, 1H). **^13^C NMR** (101 MHz, CDCl_3_, free base) δ 154.60 (s), 151.11 (s), 126.65 (s), 123.73 (s), 120.58 (s), 105.12 (s), 72.60 (s), 71.97 (s), 71.74 (s), 47.61 (s), 30.08 (s), 29.45 (s).

**2-Amino-1-(8-bromo-2,3,6,7-tetrahydrobenzo[1,2-b:4,5-b’]difuran-4-yl)ethan-1-ol (8)**

To a solution of amine **7** (177 mg, 0.8 mmol) in AcOH (4 mL), covered in aluminum foil, was added a solution of bromine (128 mg, 0.8 mmol) in AcOH (4 mL) dropwise and the reaction was stirred at room temperature for 2.5 h until full conversion of starting material (TLC; DCM/MeOH/NH_4_OH 10:1:0.01, Rf(**8**) = 0.2)). The reaction was quenched by Na_2_S_2_O_3_, diluted with DCM, and extracted with 1M HCl (3x). The aqueous phase was basified by 2M NaOH, extracted with DCM (5x), organic fractions dried over Na_2_SO_4,_ and evaporated under reduced pressure to a white solid (137 mg, 0.45 mmol, 57%). The title compound was stored as the hydrochloride salt crystallized from MeOH/Et_2_O.

**m.p.** (HCl salt) 244–245 °C; **^1^H NMR** (400 MHz, CDCl_3_, free base) δ 4.73–4.65 (m, 1H), 4.66–4.56 (m, 4H), 3.67–3.62 (m, 1H), 3.29 (t, *J* = 8.7 Hz, 2H), 3.15 (t, *J* = 8.7 Hz, 2H), 2.94 (d, *J* = 6.2 Hz, 2H).

**4-(2-Nitro-1-ethenyl)-2,3,6,7-tetrahydrobenzo[1,2-b:4,5-b’]difuran (9),**

**1-(2,3,6,7-Tetrahydrobenzo[1,2-b:4,5-b’]difuran-4-yl)-2-aminoethane (10),**

**1-(8-Bromo-2,3,6,7-tetrahydrobenzo[1,2-b:4,5-b’]difuran-4-yl)-2-aminoethane (11)**

Synthesized as previously reported [21], ^1^H and ^13^C NMR spectra in agreement with the literature [21].

**2-(8-Bromo-2,3,6,7-tetrahydrobenzo[1,2-b:4,5-b’]difuran-4-yl)-*N*-(2-methoxybenzyl)ethan-1-amine (1)**

To a solution of **11** (242 mg, 0.85 mmol) in EtOH (10 mL) under argon atmosphere, 2-methoxybenzaldehyde (127.3 mg, 0.94 mmol) was added. Upon the formation of imine (TLC) the solution was cooled with an ice-water bath and NaBH_4_ (161 mg, 4.25 mmol) was added in portions. Progress of the reaction was monitored by TLC (DCM/MeOH/NH_4_OH 10:1:0.01). After full conversion, the reaction was quenched by a dropwise addition of water, let to stir for at least 15 min., and concentrated under reduced pressure. The residue was dissolved in Et_2_O (15 mL), acidified by 1 M HCl, and extracted with water (3 × 15 mL). The aqueous phase was basified with 2 M NaOH, and extracted by DCM (5 × 15 mL). The organic phases were dried over Na_2_SO_4_ and evaporated under reduced pressure yielding the crude title compound. The substance was collected as the hydrochloride salt (311 mg, 0.71 mmol, 83%) recrystallized from MeOH/Et_2_O.

^1^H and ^13^C NMR spectra are in agreement with the literature [7].

**2-(((2-(8-Bromo-2,3,6,7-tetrahydrobenzo[1,2-b:4,5-b’]difuran-4-yl)ethyl)amino)methyl)phenol (12)**

Synthesized as (**1**) using salicylaldehyde, except using NH_4_OH instead of NaOH for basification during the workup. The hydrochloride salt of the title compound was collected as white solid (75 mg, 55%).

**m.p.** (HCl salt) 232–234 °C; **^1^H NMR** (400 MHz, CD_3_OD, HCl salt) δ 7.29–7.23 (m, 2H), 6.90–6.84 (m, 2H), 4.56 (q, *J* = 8.6 Hz, 4H), 4.21 (s, 2H), 3.24–3.16 (m, 4H), 3.13 (t, *J* = 8.7 Hz, 2H), 2.88 (t, *J* = 7.6 Hz, 2H); **^13^C NMR** (101 MHz, CD_3_OD, HCl salt) δ 157.46 (s), 152.80 (s), 132.55 (s), 132.39 (s), 128.24 (s), 127.99 (s), 121.04 (s), 118.66 (s), 116.22 (s), 114.85 (s), 99.30 (s), 72.84 (s), 72.52 (s), 48.79 (s), 48.34 (s), 46.76 (s), 32.51 (s), 30.62 (s), 25.49 (s).

**2-(((2-(8-Bromo-2,3,6,7-tetrahydrobenzo[1,2-b:4,5-b’]difuran-4-yl)ethyl)amino)methyl)-3-methoxyphenol (13)**

Synthesized as (**1**) using 2-hydroxy-6-methoxybenzaldehyde, except using NH_4_OH instead of NaOH for basification during the workup. The hydrochloride salt of the title compound was collected as a white solid (157 mg, 44%).

**m.p.** (HCl salt) decomposed; **^1^H NMR** (400 MHz, DMSO-d_6_, HCl salt) δ 10.30 (s, 1H), 8.69 (s, 2H), 7.21 (t, *J* = 8.3 Hz, 1H), 6.59 (d, *J* = 8.1 Hz, 1H), 6.55 (d, *J* = 8.2 Hz, 1H), 4.63–4.46 (m, 4H), 4.07 (t, *J* = 5.0 Hz, 2H), 3.79 (s, 3H), 3.21 (t, *J* = 8.6 Hz, 2H), 3.10 (t, *J* = 8.7 Hz, 2H), 3.04–2.94 (m, 2H), 2.87–2.77 (m, 2H); **^13^C NMR** (101 MHz, DMSO-d_6_, HCl salt) δ 158.86 (s), 157.15 (s), 152.11 (s), 150.61 (s), 130.92 (s), 127.11 (s), 126.29 (s), 114.33 (s), 108.24 (s), 105.85 (s), 101.96 (s), 97.12 (s), 71.52 (s), 70.97 (s), 55.82 (s), 44.96 (s), 40.19 (s), 31.13 (s), 29.34 (s), 24.04 (s).

**3-(((2-(8-Bromo-2,3,6,7-tetrahydrobenzo[1,2-b:4,5-b’]difuran-4-yl)ethyl)amino)methyl)-4-methoxyphenol (14)**

Synthesized as (**1**) using 2-methoxy-5-hydroxybenzaldehyde, except using NH_4_OH instead of NaOH for basification during the workup. The hydrochloride salt of the title compound was collected as a white solid (113 mg, 45%).

**m.p.** (HCl salt) 244.2–245.7 °C; **^1^H NMR** (400 MHz, DMSO-d_6_, HCl salt) δ 9.23 (s, 1H), 8.90 (s, 2H), 6.91 (d, *J* = 8.9 Hz, 1H), 6.86 (d, *J* = 2.8 Hz, 1H), 6.80 (dd, *J* = 8.8, 2.9 Hz, 1H), 4.63–4.50 (m, 4H), 4.05 (s, 2H), 3.74 (s, 3H), 3.22 (t, *J* = 8.7 Hz, 2H), 3.11 (t, *J* = 8.6 Hz, 2H), 3.05–2.96 (m, 2H), 2.86–2.78 (m, 2H); **^13^C NMR** (101 MHz, DMSO-d_6_, HCl salt) δ 152.11 (s), 150.89 (s), 150.61 (s), 150.27 (s), 127.14 (s), 126.28 (s), 120.25 (s), 118.21 (s), 116.52 (s), 114.34 (s), 112.17 (s), 97.12 (s), 71.53 (s), 70.95 (s), 55.92 (s), 45.00 (s), 44.90 (s), 31.14 (s), 29.39 (s), 24.10 (s).

**4-(((2-(8-Bromo-2,3,6,7-tetrahydrobenzo[1,2-b:4,5-b’]difuran-4-yl)ethyl)amino)methyl)-3-methoxyphenol (15)**

Synthesized as (**1**) using 2-methoxy-4-hydroxybenzaldehyde, except using NH_4_OH instead of NaOH for basification during the workup. The hydrochloride salt of the title compound was collected as a white solid (32 mg, 22%).

**m.p.** (HCl salt) decomposed; **^1^H NMR** (400 MHz, CD_3_OD, HCl salt) δ 7.17 (d, *J* = 8.2 Hz, 1H), 6.50 (d, *J* = 5.2 Hz, 1H), 6.43 (dd, *J* = 8.2, 2.2 Hz, 1H), 4.64–4.53 (m, 4H), 4.12 (s, 2H), 3.83 (s, 3H), 3.28–3.10 (m, 6H), 2.93–2.81 (m, 2H); **^13^C NMR** (101 MHz, DMSO-d_6_, HCl salt) δ 159.99 (s), 158.75 (s), 152.11 (s), 150.61 (s), 132.54 (s), 127.12 (s), 126.27 (s), 114.36 (s), 109.79 (s), 107.02 (s), 98.97 (s), 71.53 (s), 70.95 (s), 55.46 (s), 44.79 (s), 44.55 (s), 31.13 (s), 29.39 (s), 25.62 (s), 24.09 (s).

***N*-(2-(methoxy-d_3_)benzyl)-2-(2,3,6,7-tetrahydrobenzo[1,2-b:4,5-b’]difuran-4-yl)ethan-1-amine (16)**

Synthesized as (**1**) using 2-(methoxy-d_3_)benzaldehyde, 2C-H-Fly hydrochloride, and 1 mol. eq. of triethylamine (to form a free base in situ). The obtained free base of crude product (yellowish oil) was diluted with methanol and converted to hydrochloride by addition of concentrated aq. solution of HCl. The solution was evaporated to dryness and the resulting solid was recrystallized from EtOAc yielding white crystals in 35% yield.

**m.p.** (HCl salt) 203–204 °C; **^1^H NMR** (400 MHz, CD_3_OD, HCl salt) δ 7.52–7.33 (m, 2H), 7.14–6.97 (m, 2H), 6.55 (s, 1H), 4.51 (td, *J* = 8.5; 5.6 Hz, 4H), 4.26 (s, 2H), 3.24 (dd, *J* = 8.6, 6.8 Hz, 2H), 3.18–3.06 (m, 4H), 2.95 (dd, *J* = 8.6, 6.8 Hz, 2H); **^13^C NMR** (101 MHz, CD_3_OD, HCl salt) δ 159.3 (s), 155.8 (s), 153.5 (s), 132.7 (s), 132.7 (s), 127.7 (s), 126.6 (s), 122.1 (s), 120.2 (s), 115.4 (s), 112.1 (s), 106.0 (s), 79.5 (s), 72.6 (s), 72.5 (s), 55.6–55.2 (m), 48.0 (s), 47.0 (s), 31.3 (s), 29.6 (s), 25.7 (s). **HRMS-ESI**: *m/z* [M + H]^+^ calculated for C_20_H_20_D_3_NO_3_ + H^+^ (329.1939), found (329.1920).

**2-(8-Bromo-2,3,6,7-tetrahydrobenzo[1,2-b:4,5-b’]difuran-4-yl)-*N*-(2-(methoxy-d_3_)benzyl)ethan-1-amine (17)**

Synthesized as (**1**) using 2-(methoxy-d_3_)benzaldehyde. The hydrochloride salt of the title compound was collected as a white solid (155 mg, 0.35 mmol, 70%).

**m.p.** (HCl salt) 214.0–217.0 °C; **^1^H NMR** (400 MHz, CD_3_OD, HCl salt) δ 9.10 (s, 2H), 7.46 (dd, *J* = 7.5, 1.6 Hz, 1H), 7.42 (ddd, *J* = 8.3, 7.5, 1.7 Hz, 1H), 7.09 (dd, *J* = 8.3, 0.9 Hz, 1H), 7.00 (td, *J* = 7.4, 1.0 Hz, 1H), 4.56 (dt, *J* = 12.4, 8.7 Hz, 4H), 4.12 (s, 2H), 3.22 (t, *J* = 8.7 Hz, 2H), 3.09 (dd, *J* = 17.3, 8.6 Hz, 2H), 3.06–2.97 (s, 2H), 2.90–2.81 (m, 2H); **^13^C NMR** (101 MHz, DMSO-d_6_, HCl salt) δ 157.47 (s), 152.11 (s), 150.61 (s), 131.44 (s), 130.86 (s), 127.14 (s), 126.28 (s), 120.39 (s), 119.65 (s), 114.33 (s), 111.13 (s), 97.11 (s), 71.53 (s), 70.95 (s), 44.96 (s), 44.91 (s), 40.20 (s), 31.13 (s), 29.40 (s), 24.08 (s).

### 4.5. Sample Preparation

#### 4.5.1. Human Liver Microsomes

The microsomal membranes were disrupted by three consecutive freeze-thaw cycles. Then, the solid residues were separated by centrifugation (Eppendorf 5415; 13,200 rpm, 10 min, 4 °C) and filtered off. An amount of 200 μL of the liquid supernatant was transferred to an Eppendorf tube before the internal standard was added (20 μL of 1 μg·mL^−1^ solution of 2C-B-Fly-NBOMe-*d*_3_ (**17**)). Subsequently, acetonitrile was evaporated using vacuum concentrator Hanil Modul 4080C and the pH value of the samples was adjusted by the addition of NH_4_HCO_3_ solution. The resulting aqueous phase was shaken with ether (2 × 400 μL). The obtained organic extracts were combined and evaporated. The dry residue was reconstituted in 200 μL of a solvent mixture containing methanol and 0.1% aqueous formic acid in a ratio of 5:95 (*v*/*v*). The clear solution was injected from a vial equipped with an appropriate insert to LC-MS/MS system.

#### 4.5.2. *C. elegans* Culture Medium

*C. elegans* culture media were processed as described in our previous study [29]. The amount of 200 μL of the liquid medium was transferred to an Eppendorf tube before the IS was added (20 μL of 1 μg·mL^−1^ solution of 2C-B-Fly-NBOMe-*d*_3_ (**17**)). Solution of NH_4_HCO_3_ was used to adjust the pH value of the samples to approximately 8.4. The aqueous phase was shaken with ether (2 × 400 μL), and the resulting organic extracts were combined and evaporated. The dry residue was reconstituted in 200 μL of a solvent mixture of methanol and 0.1% aqueous formic acid in the ratio of 5:95 (*v*/*v*). The clear solution was automatically injected from a vial equipped with an appropriate insert to LC-MS/MS system.

#### 4.5.3. Rat Urine

Rat urine was processed using an adapted method for metabolomics studies of NPS described in our previous work [29]. An amount of 200 μL of collected urine was diluted with 200 μL of a solvent mixture of methanol and 0.1% aqueous formic acid in a ratio of 9:91 (*v*/*v*) containing IS (200 ng·mL^−1^ of 2C-B-Fly-NBOMe-*d*_3_ (17)). The solution was shortly vortexed before the formed precipitate was separated by centrifugation (Eppendorf 5415 R; 13,200 rpm, 10 min, 4 °C). The resulting clear supernatant was automatically injected from a vial equipped with an appropriate insert to LC-MS/MS system.

### 4.6. LC-MS Analysis

#### 4.6.1. Untargeted Screening

The information-dependent acquisition (IDA) analysis was performed on the UltiMate 3000 HPLC system (Thermo Fisher Scientific, Waltham, MA, USA) connected to the hybrid mass spectrometer TripleTOF 5600 (AB Sciex, Framingham, MA, USA). Ionization of analytes was achieved by the electrospray technique. Chromatographic separation was performed on a Kinetex C18 analytical column (Phenomenex, Torrance, CA, USA) with the following dimensions: 3 × 50 mm and particle size 1.7 µm. Solvent A consisted of 0.1% aqueous formic acid with 5 mM NH_4_HCO_2_ and solvent B was 0.1% methanolic solution of formic acid. The flow rate of the mobile phase was set at 200 μL/min and the following gradient elution program was applied: 0–0.3 min: 2% B, 0.3–0.4 min: 2% to 4% B, 0.4–1 min: 4% to 10% B, 1–12 min: 10% to 100% B, 12–17.6 min: 100% to 10% B, 17.6–18 min: 10% to 2% B, 18–20 min: 2% B.

All data were acquired in positive, high sensitivity mode. The setup of the apparatus was as follows: ion spray voltage floating (ISVF) 4 kV, source temperature 350 °C, curtain gas 35 psi, nebulizer (GS1) gas 30 psi, and heater (GS2) gas 40 psi. Sciex OS-Q software was used to evaluate both HR-MS and HR-MS/MS spectra acquired in the information-dependent acquisition (IDA) experiments. Both a survey and a dependent scan were measured over a mass range from 50 to 800 *m*/*z*, with collision-induced dissociation triggered by rolling collision energy.

#### 4.6.2. Fragmentation in MS^3^

The fragmentation analysis was performed on the UltiMate 3000 HPLC system (Thermo Fisher Scientific) connected to the triple quadrupole mass spectrometer QTrap 6500 (AB Sciex). Ionization of analytes was achieved by electrospray technique using a Turbo V ion source. One of the following optimized HPLC methods was chosen based on the analyte polarity. Chromatographic separation of highly non-polar standards was performed on a Poroshell 120 EC-CN analytical column (Agilent, Santa Clara, CA, USA) with the following dimensions: 2.1 × 50 mm and particle size 2.7 µm. Solvent A consisted of 0.1% aqueous formic acid with 5 mM NH_4_HCO_2_ and solvent B was acetonitrile. The flow rate of the mobile phase was set at 600 μL/min and the following gradient elution program was applied: 0–1 min: 2% B, 1–8 min: 2% to 30% B, 8–9.5 min: 30% to 50% B, 9.5–11 min: 50% to 98% B, 11–13 min: 98% B, 13–13.5 min: 98% to 2% B, 13.5–15 min: 2% B. Chromatographic separation of more polar standards was performed on a Luna Omega Polar C18 analytical column (Phenomenex, Torrance, CA, USA) with the following dimensions: 2.1 × 100 mm and particle size 3 µm. Solvent A consisted of 0.1% aqueous formic acid with 5 mM NH_4_HCO_2_ and solvent B was methanol. The flow rate of the mobile phase was set at 400 μL/min and the following gradient elution program was applied: 0–2 min: 2% B, 2–12 min: 2% to 98% B, 12–14.5 min: 98% B, 14.5–15 min: 98% to 2% B, 15–20 min: 2% B.

All data were acquired in positive ion mode. The setup of the apparatus was as follows: ion spray voltage 5350 V, source temperature 450 °C, curtain gas 20 psi, nebulizer (GS1) and heater (GS2) gases 30 psi. For data acquisition, Analyst software version 1.63 was utilized (AB Sciex).

## 5. Conclusions

In this study, the chemical structures of phase I and phase II metabolites of 2C-B-Fly-NBOMe in HLM, rat urine, and *C. elegans* mycelium were proposed based on detected HR-MS/MS spectra and their comparison with synthesized reference standards was performed. Major metabolic pathways of 2C-B-Fly-NBOMe are mono- and poly-hydroxylation, *O*-demethylation, and oxidative debromination, with several differences between the media. HLM matrix provided the highest amounts of phase I metabolites. Lesser amounts were detected in the rat urine, including two demethylated metabolites hydroxylated to higher degrees that were found only in this in vivo model. Compared to the other two media, the extent of debromination was highest in the mycelium matrix, and several dehydrogenated and *N*-oxygenated metabolites were produced. The possible differences between species should be considered in studies of the metabolism of novel substances.

## Figures and Tables

**Table 1:** 2C-B-Fly-NBOMe and its proposed metabolites: precursor ion masses, characteristic fragment ions in MS^2^ and MS^3^ scan modes, and retention times (RT). The first order product ions fragmented in linear ion trap are written in bold. Data were acquired by an UltiMate 3000 LC system (Thermo Fisher Scientific, Waltham, MA, USA) combined with a QTrap 6500 mass spectrometer (Sciex).

Synthesized Standard	Precursor Ion [*m*/*z*]	MS^2^ Fragment Ions [*m*/*z*] ^1^	MS^3^ Fragment Ions [*m*/*z*] ^1^	HPLC Method	RT [min]
2C-B-Fly-NBOMe (**1**)	404	91 (42), 121 (100), 173 (1), 188 (2), 267 (8), 296 (8), 325 (5)	**121**: 77 (9), 91 (100), 93 (94), 121 (2) **267**: 145 (3), 159 (7), 173 (24), 188 (100)	method I	6.0
C4’-hydroxy-2C-B-Fly-NBOMe (**15**)	420	107 (15), 137 (100), 188 (5), 267 (92), 284 (34)	**137**: 77 (14), 79 (21), 107 (100), 109 (16), 137 (33) **284**: 173 (2), 188 (12), 267 (100), 284 (40)	method I method II	5.1 8.8
C5’-hydroxy-2C-B-Fly-NBOMe (**14**)	420	77 (10), 107 (70), 137 (100), 188 (4), 203 (2), 267 (15), 312 (12), 341 (5), 420 (11)	**312**: 147 (16), 175 (100), 188 (11), 279 (8), 312 (47) **341**: 175 (43), 203 (6), 310 (27), 312 (78), 341 (100)	method I method II	5.2 8.8
C6’-hydroxy-2C-B-Fly-NBOMe (**13**)	420	77 (5), 107 (22), 109 (14), 137 (100), 188 (8), 267 (98), 284 (78)	**267**: 145 (3), 159 (5), 173 (22), 188 (100), 267 (74) **284**: 173 (1), 188 (7), 267 (100), 284 (4)	method II	9.3
2C-H-Fly-NBOMe-*d*_3_ (**16**)	329	91 (86), 124 (100), 176 (5), 187 (13), 189 (49), 204 (8), 312 (32), 329 (27)	**189**: 133 (9), 161 (26), 174 (17), 187 (100), 189 (23) **312**: 124 (93), 150 (53), 175 (40), 201 (73), 270 (93), 312 (100)	method I	4.2
2C-B-Fly-NBOH (**12**)	390	77 (3), 107 (64), 173 (1), 188 (13), 254 (1), 267 (100), 284 (40), 390 (12)	**267**: 145 (4), 159 (6), 173 (26), 188 (100), 267 (94) **284**: 173 (1), 188 (10), 267 (100), 284 (11)	method I	5.1
2C-B-Fly (**11**)	284	145 (1), 159 (2), 173 (9), 188 (48), 267 (100), 284 (2)	**188**: 131 (5), 145 (9), 159 (20), 173 (100), 188 (23) **267**: 145 (3), 159 (5), 173 (19), 188 (100), 267 (29)	method II	7.7
hydroxy-2C-B-Fly (**8**)	300	174 (7), 186 (11), 203 (100), 265 (6), 282 (7)	**203**: 174 (17), 186 (19), 203 (100) **282**: 174 (3), 186 (3), 203 (100)	method II	6.8
2C-H-Fly (**10**)	206	91 (7), 105 (23), 133 (53), 161 (100), 189 (93)	**161**: 91 (2), 105 (9), 133 (27), 161 (100) **189**: 105 (15), 133 (44), 161 (100), 171 (16), 189 (27)	method II	6.4

^1^ Relative intensity of a fragment ion is given in brackets [%].

**Table 2:** 2C-B-Fly-NBOMe and its detected phase I and II metabolites: precursor ion exact masses (PM), retention times (RT), and detectability in rat urine (RU), human liver microsomes (HLM), and *C. elegans* culture medium (CE). Data was acquired by an UltiMate 3000 LC system (Thermo Fisher Scientific) combined with a hybrid TripleTOF 5600 mass spectrometer (Sciex).

No.	Metabolite	PM [*m*/*z*]	RT [min]	RU	HLM	CE
M1	2C-B-Fly-NBOMe	404.0861	8.4	C	C	C
M2	C5′-hydroxy-2C-B-Fly-NBOMe	420.0810	7.8		C	C
M3	hydroxy-2C-B-Fly-NBOMe isomer 2	420.0810	6.8	I	I	I
M4	hydroxy-2C-B-Fly-NBOMe isomer 3	420.0810	7.7		I	
M5	hydroxy-2C-B-Fly-NBOMe isomer 4	420.0810	8.1		I	I
M6	2C-B-Fly-NBOH	390.0705	8.0		C	C
M7	2C-H-Fly-NBOMe	326.1756	7.6		D	C
M8	2C-B-Fly	284.0286	6.5	D	C	C
M9	hydroxy-2C-B-Fly	300.0235	4.7	I	I	D
M10	dihydroxy-2C-B-Fly-NBOMe isomer 1	436.0760	6.1	I	I	
M11	dihydroxy-2C-B-Fly-NBOMe isomer 2	436.0760	6.2		I	
M12	dihydroxy-2C-B-Fly-NBOMe isomer 3	436.0760	6.3	I		
M13	dihydroxy-2C-B-Fly-NBOMe isomer 4	436.0760	6.5	D	I	
M14	dihydroxy-2C-B-Fly-NBOMe isomer 5	436.0760	7.1	D	D	
M15	dihydroxy-2C-B-Fly-NBOMe isomer 6	436.0760	7.3		I	
M16	hydroxy-2C-B-Fly-NBOMe-*N*-oxide	436.0760	9.0		D	D
M17	trihydroxy-2C-B-Fly-NBOMe isomer 1	452.0709	6.3	I	D	
M18	trihydroxy-2C-B-Fly-NBOMe isomer 2	452.0709	6.9			D
M19	hydroxy-2C-H-Fly-NBOMe	342.1705	6.3	I	D	
M20	dihydroxy-2C-H-Fly-NBOMe	358.1654	9.8		I	
M21	trihydroxy-2C-H-Fly-NBOMe	374.1604	9.1		I	
M22	hydroxy-2C-B-Fly-NBOH isomer 1	406.0654	6.4		I	D
M23	hydroxy-2C-B-Fly-NBOH isomer 2	406.0654	7.5		I	
M24	dihydroxy-2C-B-Fly-NBOH	422.0603	5.8	I		
M25	trihydroxy-2C-B-Fly-NBOH	438.0552	4.9	D		
M26	dehydro-2C-B-Fly-NBOMe isomer 1	402.0705	8.1		I	I
M27	dehydro-2C-B-Fly-NBOMe isomer 2	402.0705	8.6	I	I	I
M28	dehydro-2C-B-Fly-NBOMe isomer 3	402.0705	9.0		I	I
M29	dehydro-hydroxy-2C-B-Fly-NBOMe	418.0654	6.9	D	I	D
M30	dehydro-2C-B-Fly-NBOMe-*N*-oxide	418.0654	10.0			I
M31	dehydro-dihydroxy-2C-H-Fly-NBOMe	356.1498	10.0		I	
M32	dehydro-2C-B-Fly-NBOH	388.0548	8.3		I	D
M33	dehydro-dihydroxy-2C-H-Fly-NBOH isomer 1	342.1341	9.4		I	
M34	dehydro-dihydroxy-2C-H-Fly-NBOH isomer 2	342.1341	9.8		I	
M35	dihydroxy-2C-B-Fly	316.0184	4.7	D		
M3-G	hydroxy-2C-B-Fly-NBOMe glucuronide isomer 1	596.1131	6.1	I		
M4-G	hydroxy-2C-B-Fly-NBOMe glucuronide isomer 2	596.1131	6.4	I		
M9-G	hydroxy-2C-B-Fly glucuronide	476.0556	4.1	I		
M11-G	dihydroxy-2C-B-Fly-NBOMe glucuronide	612.1080	5.9	D		
M19-G	hydroxy-2C-H-Fly-NBOMe glucuronide	518.2026	5.6	D		
M22-G	hydroxy-2C-B-Fly-NBOH glucuronide isomer 1	582.0975	5.8	I		
M22-G	hydroxy-2C-B-Fly-NBOH glucuronide isomer 2	582.0975	5.9	I		
M-Ac1	dehydro-2C-B-Fly acetate	324.0235	6.4	I		
M-Ac2	dehydro-dihydroxy-2C-B-Fly acetate	356.0134	4.7	I		

C—confirmed by comparison with synthesized reference standard; I—tentatively identified; D—detected.

## Data Availability

All data are provided in the manuscript and the Supplementary Materials.

## Supplementary Materials

The following is available online at https://www.mdpi.com/article/10.3390/metabo11110775/s1, Table S1: List of 2C-B-Fly-NBOMe phase I and phase II metabolites with recorded precursor ion exact mass, the corresponding characteristic fragment ions with the calculated exact masses, elemental compositions, deviations of the measured from the calculated masses, and relative abundances of the fragment ions in HR-MS/MS.
